# Supplementary material for: CdSSe Nano-Flowers for Ultrasensitive Raman Detection of Antibiotics
Source: Molecules. 2023 Mar 27;28(7):2980. doi: 10.3390/molecules28072980 (PMC10096218; doi:10.3390/molecules28072980)
Supplement: Supplementary file 1 [file molecules-28-02980-s001.zip › molecules-2277242-supplementary.pdf]

Supplementary Information (SI)

CdSSe Nano-Flowers for Ultrasensitive Raman Detection of Antibiotics

Kangkang Wang [1,2], You Li [1,2], Honggang Wang [1,2], Ziyue Qian [1,2], Xiaokai Zhu [1,2], Sabir Hussain [1] and Liming Xie [1,2,*]

[1] CAS Key Laboratory of Standardization and Measurement for Nanotechnology, National Center for Nanoscience and Technology, Beijing 100190, China; wangkk2020@nanoctr.cn (K.W.); d202110184@xs.ustb.edu.cn (Y.L.); wanghg2020@nanoctr.cn (H.W.); qianzy2019@nanoctr.cn (Z.Q.); zhuxk2020@nanoctr.cn (X.Z.); sabirphys@yahoo.com (S.H.)

[2] School of Nanoscience and Technology, University of Chinese Academy of Sciences, Beijing 100049, China

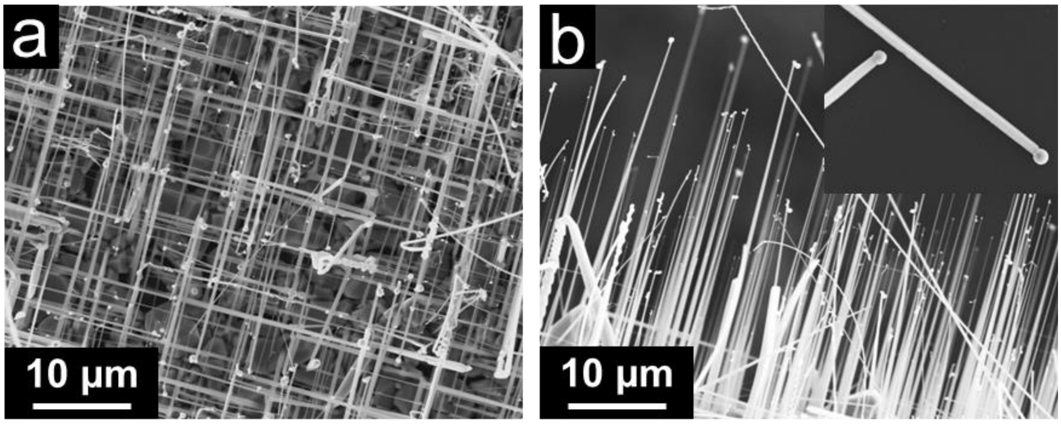

Figure S1. (a)–(b) SEM images of CdSSe NWs (inset of Figure S1b shows the single nanowire).

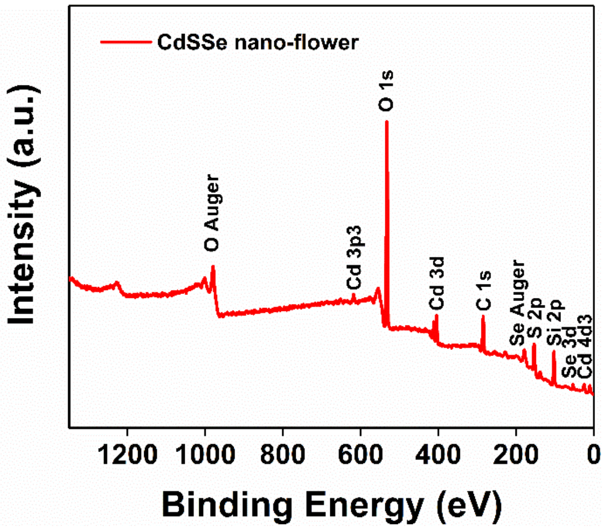

Figure S2. Wide-scan survey XPS spectrum of CdSSe NFs.

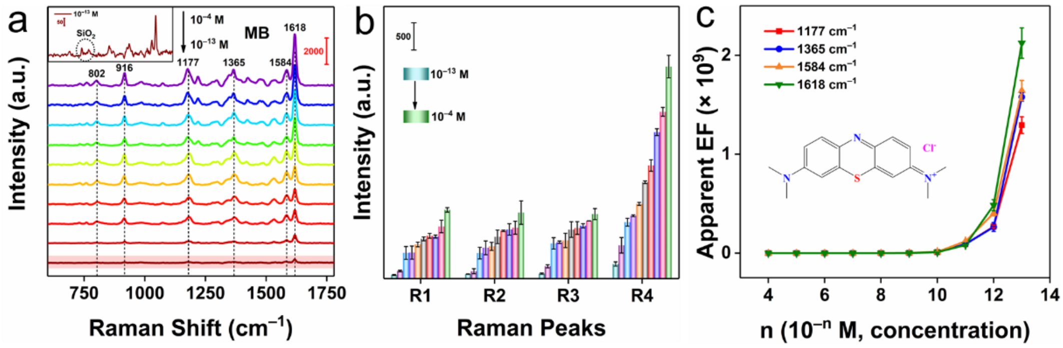

Figure S3. (a) SERS spectra, (b) statistical histogram of peak intensity (R_1 to R_4: 1177, 1365, 1584, and 1618 cm^{-1}), and (c) apparent EF values of MB solutions at varying concentrations.

Table S1. Apparent EFs of the Raman peaks of Rh6G and MB at different concentrations.

Concentration	Rh6G (cm^{-1})				MB (cm^{-1})			
(mol/L)	1184	1362	1573	1650	1177	1365	1584	1618
10^{-4}	6.15	5.34	5.37	5.60	24.29	23.60	21.21	30.78
10^{-5}	45.54	41.69	39.24	44.13	184.18	175.64	166.15	242.21
10^{-6}	408.21	358.72	352.52	350.74	1.49×10^3	1.82×10^3	1.61×10^3	2.13×10^3
10^{-7}	3.78×10^3	3.35×10^3	3.34×10^3	3.40×10^3	1.20×10^4	1.15×10^4	1.16×10^4	1.64×10^4
10^{-8}	1.17×10^4	9.91×10^3	1.14×10^4	9.42×10^3	1.51×10^5	1.71×10^5	1.90×10^5	1.40×10^5
10^{-9}	1.08×10^5	9.25×10^4	9.91×10^4	8.58×10^4	1.40×10^6	1.49×10^6	1.74×10^6	1.09×10^6
10^{-10}	5.87×10^5	5.31×10^5	5.58×10^5	4.19×10^5	9.19×10^6	1.10×10^7	1.25×10^7	9.12×10^6
10^{-11}	6.33×10^6	5.21×10^6	5.68×10^6	4.14×10^6	9.07×10^7	9.09×10^7	1.20×10^8	8.19×10^7
10^{-12}	3.30×10^7	2.25×10^7	2.65×10^7	2.17×10^7	2.69×10^8	2.64×10^8	4.03×10^8	4.84×10^8
10^{-13}	5.18×10^8	2.59×10^8	2.88×10^8	1.83×10^8	1.29×10^9	1.58×10^9	1.64×10^9	2.12×10^9
10^{-14}	3.62×10^9	1.82×10^9	1.84×10^9	1.79×10^9	–	–	–	–

Table S2. Raman intensities (I_{Raman}) of pure Rh6G (10^{-3} M) and MB (10^{-3} M), and SERS intensities (I_{SERS}) of some peaks of Rh6G and MB at different concentrations.

Intensity (a.u.)	Rh6G (cm^{-1})				MB (cm^{-1})			
	1184	1362	1573	1650	1177	1365	1584	1618
**Pure (10^{-3} M)**	3021.97	10601.48	7261.61	11081.59	1039.74	1028.09	1117.89	2530.50
**10^{-4} M**	929.30	2828.11	1948.84	3102.55	1262.64	1212.89	1185.39	3894.64
**10^{-5} M**	688.17	2209.75	1424.60	2445.41	957.49	934.31	1059.75	3064.50
**10^{-6} M**	616.80	1901.46	1279.94	1943.40	772.39	902.89	970.84	2690.38
**10^{-7} M**	571.73	1774.06	1211.07	1882.05	786.20	878.23	928.67	2077.45
**10^{-8} M**	176.52	525.19	412.88	522.11	730.23	767.26	901.37	1769.48
**10^{-9} M**	163.57	490.40	359.85	475.14	626.40	592.01	700.77	1373.67
**10^{-10} M**	95.66	281.48	202.72	232.21	477.90	564.51	668.09	1154.46
**10^{-11} M**	88.65	276.18	206.38	229.56	471.74	467.47	647.75	1035.90
**10^{-12} M**	78.29	137.20	104.41	119.96	139.59	135.62	225.05	612.91
**10^{-13} M**	54.65	119.33	96.33	101.62	67.27	81.10	91.89	268.61
**10^{-14} M**	49.88	96.57	66.96	99.36	–	–	–	–
